# d-Amino Acids in Plants: Sources, Metabolism, and Functions

**DOI:** 10.3390/ijms21155421

**Published:** 2020-07-30

**Authors:** Üner Kolukisaoglu

**Affiliations:** Center for Molecular Biology of Plants (ZMBP), University of Tübingen, Auf der Morgenstelle 32, 72076 Tübingen, Germany; uener.kolukisaoglu@zmbp.uni-tuebingen.de; Tel.: +49-7071-29-73095

**Keywords:** d-amino acids, amino acid transport in plants, amino acid metabolism, d-Met stimulated ethylene synthesis, peptidoglycan in chloroplasts

## Abstract

Although plants are permanently exposed to d-amino acids (d-AAs) in the rhizosphere, these compounds were for a long time regarded as generally detrimental, due to their inhibitory effects on plant growth. Recent studies showed that this statement needs a critical revision. There were several reports of active uptake by and transport of d-AAs in plants, leading to the question whether these processes happened just as side reactions or even on purpose. The identification and characterization of various transporter proteins and enzymes in plants with considerable affinities or specificities for d-AAs also pointed in the direction of their targeted uptake and utilization. This attracted more interest, as d-AAs were shown to be involved in different physiological processes in plants. Especially, the recent characterization of d-AA stimulated ethylene production in *Arabidopsis thaliana* revealed for the first time a physiological function for a specific d-AA and its metabolizing enzyme in plants. This finding opened the question regarding the physiological or developmental contexts in which d-AA stimulated ethylene synthesis are involved in. This question and the ones about the transport characteristics of d-AAs, their metabolism, and their different physiological effects, are the focus of this review.

## 1. Introduction

It is a well-established fact that proteinogenic L-amino acids are key molecules of life as the building blocks of proteins, but also as intermediates and final products of primary metabolism. In contrast, their enantiomers were for a long time regarded as useless, or even harmful for plants. This is puzzling, as d-AAs fulfill important or even essential functions in organisms from almost any other kingdom. The most prominent example in this regard is the composition of the bacterial cell wall, the peptidoglycan. This molecule consists of polysaccharide chains, which are linked by oligopeptides. These oligopeptides contain a high proportion of d-AAs, especially d-Ala and d-Glu [1]. Apart from its structural properties as a bacterial exoskeleton, resistant against peptidolytic degradation, the elements of the peptidoglycan layer are released as muropeptides, for interaction with other different organisms, ranging from bacteria to humans (for a summary see [2]).

In the animal kingdom, many examples of d-AAs fulfilling physiological functions are described. They are regularly found in bioactive peptides of invertebrates, amphibians, and reptiles. These peptides are proposed to be synthesized non-ribosomally, acting as antibiotics, hormones, or venoms (for summaries see [3,4,5]). However, d-AAs in lower and higher animals are predominantly found in their free forms and the significance of these compounds for animal and especially human physiology is a matter of intensive research. For instance, d-Asp and d-Ser bind to the NMDA (N-methyl-d-aspartate) receptor, an ionotropic glutamate receptor at excitatory synapses regulating a number of neurophysiological functions. Both d-AAs were shown to regulate NMDA receptor activity, so it is not astonishing that an imbalance of these particular d-AAs in different body fluids are discussed to cause various neurological and psychiatric disorders, like Alzheimer′s disease and schizophrenia [6]. Therefore, several d-AAs are also administered to counteract these effects [6,7]. However, there are more d-AAs found in animals than just d-Asp and d-Ser. Recently, 12 different free d-AAs could be identified in the colon of mice produced by commensal bacteria [8], but their functions in the interplay of the bacteria with their hosts are still controversial.

As indicated above, the investigation on d-AA function in plants is still in its infancy, because these compounds were regarded as detrimental for plant growth and development and, therefore, were outside of research interest. Different findings in the last decade, however, revealed that this point of view needed to be revised. d-AAs turned out to be the subject of transport processes in plants, especially in roots, which is surrounded by soil containing d-AAs as a major nutritional source. Additionally, plant genomes contain genes encoding different enzymes putatively specific for d-AAs metabolization. These observations match with reports about de novo synthesis of particular d-AAs in plants. Finally, d-AAs were found to be utilized as nitrogen source by plants, but also to affect chloroplast division and to stimulate ethylene production. Examples and evidences for these d-AA related phenomena in plants will be summarized and discussed here. In conclusion, plants seem to have adopted different d-AAs as nitrogen source, building blocks, or signaling molecules. Number and diversity of d-AA functions indicate their relevance for the plants´ physiology.

## 2. d-AA Transport in Plants: They Get In and They Get Out, but How?

Amino acids are the major source of organic nitrogen for plants [9] and in some cases the d-enantiomer can represent more than 50% of an individual amino acid in the soil (for a summary see [10]). Therefore, roots are permanently challenged by d-AAs in the soil, which mainly evolve due to chemical racemization and bacterial colonization. Exogenous application of different d-AAs to growth media affects the growth and development of different plant species negatively, as well as positively, depending on the applied d-AA [11,12,13,14]. This impact of D-AAs implies their uptake by the root, which was demonstrated in *Arabidopsis* and wheat [14,15,16,17].

**Figure 1:**
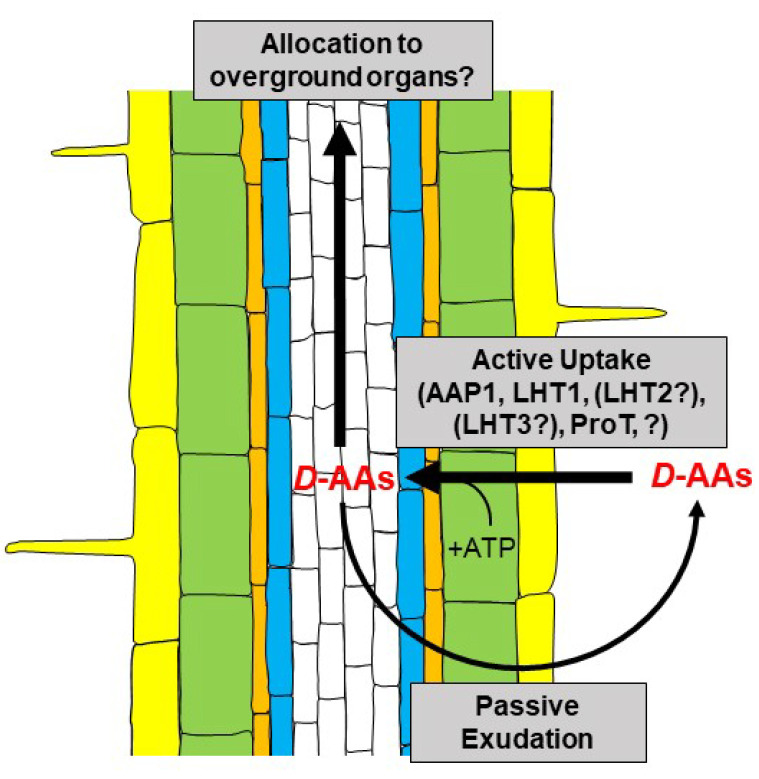
Ways of d-AA allocation in the root.

These observations raised the question about the transport mechanisms leading to the D-AA uptake by plants. In 2006, Hirner et al. [18] characterized LHT1 (Lysine Histidine Transporter 1) as a major contributor to the uptake of amino acids by roots. Svennerstam and colleagues [19] were able to show that this transporter is also responsible for the uptake of d-Ala. Later, *lht1* mutants revealed decreased uptake rates of many more d-AAs [14]. These observations led to the conclusions that LHT1 is responsible for d-AA uptake from soil. However, there must also be other proteins contributing to this transport, as exemplified in Figure 1. By overexpression of *LHT2* and *LHT3* in a *lht1* mutant, it could be unraveled that other members of the LHT family are also able to transport d-AAs, but their involvement in d-AA root uptake is not yet resolved [20]. AAP1 (Amino Acid Permease 1) is another broad-range transporter involved in amino acid uptake [21], which was shown to be involved in the uptake of d-Met and d-Phe [22], but not its homolog AAP5 [20]. Regarding the Pro transporter family ProT, it was shown that all its members facilitate the uptake of *L*-, as well as d-Pro in *Arabidopsis* [23]. These reports revealed mixed affinities of different amino acid transporters not exclusive to *L*-AAs, but also to d-AA. Such properties were also observed for amino acid transporters from animals [24,25,26].

These examples give an impression about the uptake capacity of plants d-AAs, and the cited studies imply that even more transporter proteins are involved in this process waiting to be unraveled. Nevertheless, the uptake is just the first step of d-AA shuttling in plants. A possible (and plausible) next step would be the consecutive transport to leaves and other above-ground organs. Although such transport was shown for *L*-AAs (for a summary see [27]), studies of a d-AA transport to leaves are still missing. In this case, the loading and unloading of the phloem would be of special interest.

Instead, root exudation of d-AAs as reversal of the uptake process could be observed and characterized, recently. Root exudation of *L*-AAs is a well-known phenomenon and there is growing evidence for its influence on microbial colonization of the rhizosphere (for summaries see [28,29,30]). Recently, it was demonstrated that roots of *A. thaliana* are also able to exude d-AAs to their environment. Interestingly, this outward directed transport seemed to be passive, which also holds true for *L*-AAs, whereas the uptake was ATP driven [31]. This means that there is a two-way flux of d-AAs consisting of active uptake by the root and diffusion out of it. However, it was also shown that the leaking d-AAs undergo reuptake (Figure 1). The result of both processes are cycles of passive efflux and active influx. The balance of *L*-AAs, especially the proportion of exudation, is a determinant of soil quality and microbial rhizosphere colonization [32,33,34,35,36]. A central question at this point is whether the d-AA homeostasis in the rhizosphere also contributes to its microbial colonization and to the composition of its community. One point of interest in this respect would be which additional transporters contribute to the differential root uptake and exudation profiles of d-AAs. In previous studies, some members of the ABC (ATP Binding Cassette) transporter family were identified to affect exudation of *L*-AAs with significant effects on microbial root colonization [35,37,38]. Therefore, it would be interesting to find out whether members of this large plant transporter family [39] are also involved in d-AA-mediated effects in the rhizosphere.

## 3. D-AA Metabolism in Plants: Many Ways to Handle

The aforementioned study of Hener et al. [31] also revealed that the contribution of d-AA root exudation to the homeostasis of these compounds within the plants is relatively small, and can be neglected in this regard. Therefore, the question remained as to how plants further process d-AAs after being taken up. By feeding *Arabidopsis* plants with particular d-AAs it was shown that they seem to be converted to their *L*-enantiomers, pointing to AA racemization. However, exogenous application of any tested d-AA in almost all cases resulted in the formation of d-Ala and d-Glu [15]. This led to the hypothesis that the formation of d-Ala and d-Glu is driven by a d-AA specific transaminase [10,15]. Recently, we confirmed this assumption by the characterization of a previously identified transaminase [40], which contributes to the metabolization of d-AAs in *Arabidopsis* [41]. We could show in this study that loss-of-function mutants of this gene, *AtDAT1*, almost completely lose the ability to move the amino group of any given d-AA to either pyruvate or 2-oxoglutarate (2-OG), leading to d-Ala and d-Glu, respectively (Table 1). Simultaneously, these mutants lose the ability to form the corresponding *L*-enantiomers from any d-AA. That the formation of the *L*-enantiomers was not due to racemization was postulated before [15], as the transamination also results in free keto acids (Table 1), which are subsequently aminated by *L*-AA specific transaminases.

The transamination via AtDAT1 seems to be the initial major step in the utilization of most d-AAs. However, as the products of this enzyme are again d-AAs (d-Ala and, to lesser extent, d-Glu), the question remained as to how d-Ala and d-Glu are processed further. Previous studies revealed that after feeding of d-Ala and d-Glu, seedling levels of both d-AAs decrease comparably in *dat1* mutants and the corresponding wild-type plants [31]. As in this experiment, the concentration of *L*-Ala and *L*-Glu increased reciprocally in mutants, and wild-type lines racemization seemed likely. As shown in Table 1, there are several characterized candidate enzymes in plants, for the subsequent utilization of d-Ala and d-Glu and other d-AAs. However, the available data, as represented in Table 1, also make it clear that none of the yet characterized racemases shows sufficient affinity to d-Ala or d-Glu.

One explanation for this discrepancy might be that the corresponding racemases are not yet identified, and therefore, do not appear in Table 1. Plant proteins with Ala racemase activity were isolated and characterized biochemically [54,55], but they could not be identified yet. As putative candidates, there are two genes in the *Arabidopsis* genome encoding for proteins with similarity to bacterial alanine racemases, awaiting to be characterized [22]. When it comes to glutamate racemization, there is another gene in the *Arabidopsis* genome that codes for an enzyme with similarities to the members of the Asp-Glu racemase family. However, it was previously shown that these plant proteins exhibit neglectable affinity and activity towards Glu as a substrate [46].

Despite the (potential) existence of amino acid racemases in plants, there are other possibilities for the decrease of the d-Ala and d-Glu amounts in plants. As the reciprocal increase and decrease of *L*-AA and d-AA levels, respectively, was not shown by feeding plants labeled d-AAs, it could not be accounted for as a direct evidence for racemization. Therefore, this effect might also be caused by a secondary reaction, so other ways of decrease like degradation, condensation, or conjugation processes must be considered. One possible degradation mechanism could be the oxidation of d-AAs by a d-AA-specific oxidase, as listed in Table 1. Although such an activity has not been detected in plants before [56], d-AA oxidase activity of a recombinant enzyme from corn was characterized. In addition, genes coding for orthologs of such enzymes are found in the genomes of other plants like rice and *Arabidopsis* [48]. d-Glu was not tested as substrate in this study, but d-Ala was oxidized by this enzyme. Another way of reduction of d-AA amount in plants, specifically for d-Ala, might be its dimerization, which was reported several years ago [57]. Although the responsible enzyme, a d-Ala-d-Ala ligase (DDL), was functionally characterized in *Physcomitrella* and *Arabidopsis* [53], its contribution to d-Ala metabolism has not yet been investigated. There are reports of d-Ala dimerization also with other amino acids [58,59], but the corresponding enzymes that could catalyze such a reaction, are not known. The third way to control the d-AA reduction, which was described before, is malonylation. The malonylation of d-AAs is not confined to d-Ala but was also reported for other d-AAs [60,61]. This topic is discussed in the next section. Nevertheless, also in this case, the corresponding enzymes still await discovery.

The catabolism of d-Ala and d-Glu in plants is an example for the wide range of putative enzymes involved in this process. Moreover, it illustrates how limited our knowledge of this metabolic pathway still is. The range and number of ways to metabolize d-AAs raise the question, as to why plants developed and maintained such an enzymatic machinery. The central question at this point is whether d-AAs in plants are regarded as xenobiotic compounds, as a resource of organic nitrogen, or fulfil specific functions in the physiology of plants.

## 4. Physiological Functions of d-AAs in Plants: Are D-AAs Just Another Source of Nitrogen, or More?

As mentioned in Section 2, particular d-AAs can have an inhibitory effect on seedling growth, but there is growing evidence also of promoting effects of d-AAs on plant growth. Just recently, it was demonstrated that combinatory application of 0.1 mM d-Leu, d-Val, and d-Cys leads to enhanced growth of pepper plants [11]. It was also shown that feeding of 1-10 mM d-Lys and d-Ile to *Arabidopsis* seedlings resulted in better growth than their L-enantiomers at the same concentrations (Kolukisaoglu, unpublished results). The growth promoting effects could be explained either by growth stimulatory effects or by utilization of d-AAs as a nitrogen source or both. Hill et al. [16] provided evidence for the uptake and assimilation of d-Ala in wheat, comparable to the rate of its proteinogenic *L*-enantiomer. This was the first evidence for d-AAs being a bioavailable organic nitrogen source for plants. In contrast, d-Ala also inhibits the growth of *Arabidopsis* seedlings at concentrations above 0.5 mM [12,14]. As in the case of wheat, just the uptake rates were measured. This putative contradiction needs to be solved through comparable experiments. Another conclusion drawn from all these studies might be that d-AAs must not be regarded as a class of compounds with homogenous properties, but each d-AA must be assessed individually.

Apart from the effects of exogenously applied d-AAs on plant growth and development, there were early reports of de novo synthesis of d-AAs [62] and amino acid racemase activities, in different plant species. Some of the respective enzymes were later identified at the molecular level (see Table 1). Altogether, these findings showed that plants are able to produce d-AAs themselves and implied a function for these compounds in plants apart from just being an organic nitrogen source. One of the first substantial reports in this direction was published by Michard et al. [63]: The authors showed that the pollen growth in the pistil of *Arabidopsis* is regulated by d-Ser. The explanation for this effect was given by the involvement of members of the Glutamate Receptor (GLR) protein family in this process. In animals, the most prominent members of this family are the NMDA receptors (N-methyl-d-aspartate), which are essential for neurotransmission in the mammalian brain. NMDA receptors bind d-Ser as a co-agonist, and concentration and binding of this d-AA at the modulatory site of NMDAR is a major cause of schizophrenia in the human brain [64,65].

**Figure 2:**
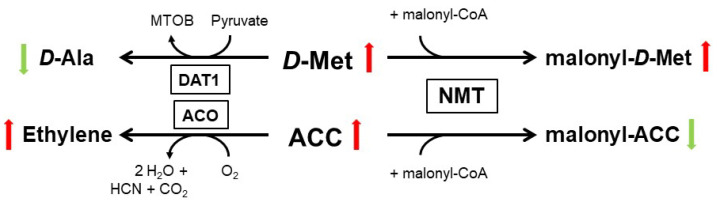
Major metabolic pathways of d-Met and ACC in *Arabidopsis*, according to [41]. Green and red colored arrows indicate the decrease and increase of both educts and their products, respectively, in the course of d-Met accumulation, as it happens in plants with loss of DAT1 activity. The ACO reaction was according to [70]. ACO—ACC oxidase; NMT—N-malonyl-transferase.

The regulation of pollen growth by d-Ser was the first example of a d-AA as a signaling molecule in plants. Another example for such a function of a d-AA was provided by the physiological role of d-Met in *A. thaliana*. Almost forty years ago, it was reported that application of d-Met and other particular d-AAs lead to increased cellular ethylene concentration in several plant species [66,67]. As we recently characterized the substrate affinity of AtDAT1, we observed a preference toward d-Met (Table 1). Additionally, *dat1* mutants remained small when germinated on d-Met, due to enhanced ethylene synthesis, in comparison to the wild type. Further analyses revealed that AtDAT1 is responsible for the decrease of d-Met content by transamination in *Arabidopsis* plants. The loss of this gene leads to an increased malonylation of d-Met and a reciprocal decrease of malonyl-ACC, the major product of the inactivation of the precursor of ethylene, ACC (1-aminocyclopropane-1-carboxylic acid), as shown in Figure 2 [41]. In this case, a d-AA (d-Met) modulates the production of a hormone, and when the activity of the central processing enzyme is lost, the high level of d-Met outcompetes ACC to be malonylated by the N-malonyl-transferase (NMT). Finally, the excess ACC becomes oxidized to ethylene. Most interestingly, the NMT, which seems to be responsible for inactivation of the majority of excessive ACC and thereby to control cellular ethylene concentration, was postulated decades ago but could not be identified till date [68,69].

Also d-Cys has a large impact on different developmental processes, in this case as the precursor of the gaseous transmitter H_2_S (for a summary on H_2_S in plants refer to [71]). One of the responsible enzymes for the production of this gas is the d-Cys desulfhydrase (d-CDes), which converts d-Cys to pyruvate, NH_3_, and H_2_S (Table 1). Isoforms of this enzyme were identified and characterized in different plant species [50,51,52]. H_2_S regulates several developmental processes, especially stress responses [71,72,73]. Recently, Zhang et al. [74] provided evidence that specifically H_2_S produced by d-CDes catalysis mediates Cd tolerance in *A. thaliana*. As this protein and its isoforms were conserved in their d-Cys desulfhydrase activity, it is tempting to speculate which other stress responses are also under its control.

A recent publication about d-Ala in the plastidic envelope of a moss [53] opened the discussion if d-AAs might also function as structural elements in plant plastids. According to classical endosymbiont hypothesis, land plant chloroplasts originated from cyanobacteria, which possessed a cell wall consisting of peptidoglycan, with a high proportion of d-Ala and d-Glu. Therefore, it was a rather intuitive step to ask whether chloroplasts still inherit parts of peptidoglycan in their envelope. This assumption was supported by the previous observation that the genome of the bryophyte *Physcomitrella patens* encodes for homologs of almost all ten bacterial genes needed for the biosynthesis of peptidoglycan [75]. Later, it was shown that this genome contains homologs of all these ten genes [76]. In a combination of genetic and physiological approaches, Hirano et al. [53] provided evidence that the chloroplast envelope of *Physcomitrella* contains the dipeptide d-Ala-d-Ala, as it is found in the bacterial peptidoglycan. In this study, a loss-of-function mutant of the *Physcomitrella ddl* gene (Table 1) revealed to be defective in plastid division, whereas the loss-of-function mutant of the orthologous gene in *Arabidopsis* remained unaffected. Due to this observation, it was concluded that peptidoglycan biosynthesis and its integration into plastidic envelopes got lost during the evolution of land plants, after the stage of lycophytes [77,78].

There were also legitimate doubts raised recently that peptidoglycan biosynthesis was really lost in the evolutionary lineage leading to seed plants [78]. It is noteworthy, that the only experimental evidence to date for the loss of peptidoglycans in higher plant chloroplasts is the observation of phenotypically unaffected chloroplasts in *Arabidopsis ddl* loss-of-function mutants (see above). Most interestingly, in RT-PCR experiments, we were able to detect a transcript in both mutants, encoding at least for the translation of a partial DDL protein in *Arabidopsis* (Hummel and Kolukisaoglu, unpublished results). This reopened the question about peptidoglycan biosynthesis in higher plants, as well. This hypothesis was supported by even more lines of evidence—the phenomenon of disturbed chloroplast division and macrochloroplasts after treatment with ß-lactam antibiotics, which specifically target peptidoglycan synthesizing enzymes, was only yet demonstrated for cryptogamic plants [79,80,81]. However, there are a number of studies also reporting the effects of these antibiotics on different developmental aspects in higher plants [82,83,84,85]. The putative lack of peptidoglycans in chloroplasts of seed plants was explained by the loss of several peptidoglycan biosynthesis gene homologs in the *Arabidopsis* genome, where just four of them were found [75]. Interestingly enough, the complete set of these genes is still found in the genomes of the gymnosperms *Picea abies* and *Pinus taeda* [86]. Further analyses of several angiosperm genomes revealed that many species retained at least the same four genes of the peptidoglycan biosynthesis in their genomes, as those found in the *Arabidopsis* genome. Intriguingly, almost the full set of genes required for peptidoglycan biosynthesis was identified in the genomes of other angiosperm species [76]. Thus, the conservation of the full set of these genes appears to have evolved in different plant lineages independently, such as apple, wine, tomato, and cocoa. However, in all these cases the last enzyme in the biosynthetic pathway, PBP (penicillin binding protein) that crosslinks peptidoglycan strands, is lacking. Instead, the authors identified in all these higher plant genomes with all peptidoglycan biosynthesis genes a novel, yet uncharacterized, gene. It encodes for a protein with a LysM domain, which the authors designated as “Peptidoglycan Pathway Associated Streptophyte Protein” (PPASP). It is intriguing to speculate that PPASP might serve as an alternative to PBP, which is to be confirmed in future. In conclusion, it must be stated that the question remains open whether chloroplasts with peptidoglycan containing envelopes are confined to cryptogamic plants or are also found in higher plants. If such chloroplasts are found in seed plants, it would be highly interesting to which extent peptidoglycan harboring chloroplasts are distributed among these organisms: Are they just found in gymnosperms with a full set of peptidoglycan biosynthesis genes like in *Physcomitrella*, are they also found in angiosperms with a quasi-full set of these genes in their genome, or are they found even in all higher plants?

## 5. Conclusions: Open Questions Galore

According to the knowledge gathered about transport, metabolism, and physiology of d-AAs in plants, which was described in the previous chapters, it becomes obvious that plants developed specific mechanisms during their evolution to cope with these compounds in the rhizosphere. As indicated in Section 2, there are still a number of unresolved questions, when it comes to the uptake and exudation of d-AAs, as well as d-AA allocation within the plant. Especially exudation is an interesting aspect against the background that certain bacteria are able to utilize different d-AAs as sole nitrogen and carbon sources [87] and that root exudation influences soil quality [32]. Additionally, metabolism of d-AAs in plants is not fully understood yet, as several d-AA synthesizing and catabolizing enzymes are “postulations” and still await molecular and biochemical identification.

**Figure 3:**
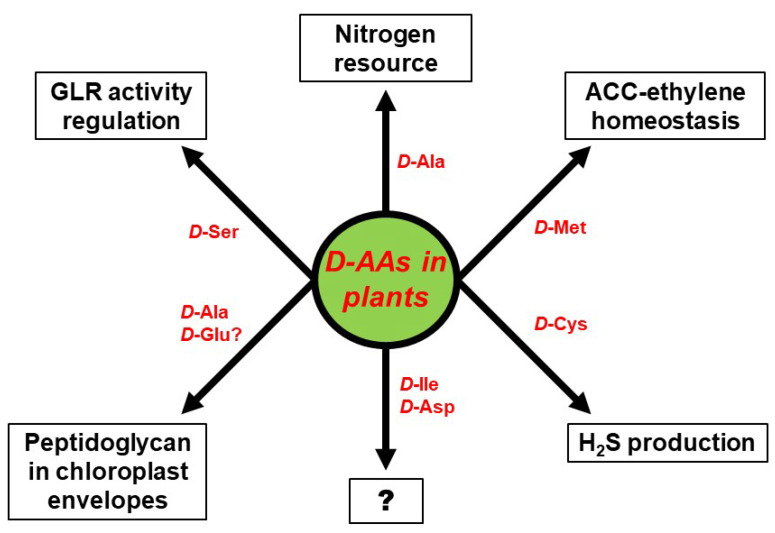
Proven and putative physiological functions of particular d-AAs in plants. The question mark (?) stands for yet unknown functions.

The biggest unresolved complex with regard to d-AAs in plants, concerns their physiological role. d-AAs appear to not just be an organic nitrogen resource but also to exert specific functions that have to be assessed in future. Some of these functional aspects are summarized in Figure 3. As described above, the utilization of d-Ala as a nitrogen resource could be confirmed in wheat [16]. However, d-Ala was also shown to be part of peptidoglycan found in the envelope of moss chloroplasts [53] and could also be an integral part of higher plant chloroplasts. Furthermore, it would be interesting to elucidate whether d-Glu, which is another common intermediate of d-AA catabolism in plants [14,15], also integrates into chloroplast envelopes, as it is usually found in bacterial peptidoglycan [1]. Apart from d-Ala other d-AAs were also identified to be involved in the developmental processes in plants—d-Cys was shown to be the precursor of the gasotransmitter H_2_S [51], and d-Met is involved in the regulation of the cellular content of the gaseous hormone ethylene [41]. Activation of NMDA receptors by d-Ser in animals was reported for plants, as it was shown that pollen tube growth depends on AtGLR1 activity, which is regulated by d-Ser [63].

All these reports revealed more physiological and developmental functions of d-AAs in plants than expected before. A glance at Figure 3 also unravels far more knowledge gaps awaiting to be filled in this regard. As described above, plants are able to synthesize d-Ile and and d-Asp de novo [46,47], but the function of these d-AAs in plants is still cryptic. There is more known about the impact of d-Met and d-Cys on ethylene and H_2_S production, but both d-AAs are not detectable in soil. The fact that d-Met is released in millimolar concentrations by several bacterial species as a signal for biofilm dispersal [88], might point to a role of this d-AA in plant–microbe interaction, which has to be investigated in the future. d-Ser co-activates plant GLRs and regulates pollen tube growth. The question remains whether this is the only developmental process to be under the control of this class of proteins. There are 20 GLR encoding genes in the *Arabidopsis* genome [89] and several of them are able to bind d-Ser [90]. It can be expected that further physiological phenomena are under the control of GLRs and, thus, d-Ser (for a review see [91]). In conclusion, the research on d-AAs in plants is an emerging field with a lot of open questions worth to be addressed in the future.

## Figures and Tables

**Table 1:** Characterized d-AA utilizing and producing enzymes in plants

Proteins	EC No.	Reactions	Substrates	References
AtDAT1	2.6.1.21	d-AA + pyruvate/2-OG → Keto acid + d-Ala/d-Glu (d-AA transamination)	d-Met (preferred) and several other d-AAs	[40,41]
Ser racemases (SerR) ^1^	5.1.1.10	*L*-Ser → d-Ser/d-Ser → *L*-Ser (Ser racemization) d-Ser/*L*-Ser → Pyruvate + NH_3_ (Ser dehydration)	d- and *L*-Ser	[42,43,44,45]
Asp racemases (AspR) ^2^	5.1.1.10	*L*-Asp → d- Asp/d- Asp → *L*- Asp (Asp racemization)	d- and *L*-Asp	[46]
AtDAAR1 + AtDAAR2	5.1.1.10	*L*-Ile → d-Ile (Ile racemization)	*L*-Ile	[47]
ZmDAAO	1.4.3.3	d-AA + H_2_O + O_2_ → Keto acid + NH_3_ + H_2_O_2_ (d-AA oxidation)	d-Ala, d-Asp	[48]
d-CDes ^3^	4.4.1.15	d-Cys + H_2_O → Pyruvate + H_2_S + NH_3_ (d-Cys desulfhydration)	d-Cys	[49,50,51,52]
d-Ala-d-Ala ligase ^4^	6.3.2.4	2 d-Ala + ATP → d-Ala-d-Ala + ADP + P_i_	d-Ala	[53]

^1^ characterized from *Arabidopsis thaliana, Hordeum vulgare*, *Oryza sativa*, *Zea mays*, *Medicago truncatula*, *Manihot esculenta*, *Solanum lycopersicum*; ^2^ characterized from *Medicago truncatula*, *Manihot esculenta*, *Solanum lycopersicum*, *Sphagnum girgensohnii*, *Spirogyra pratensis*; ^3^ characterized from *Arabidopsis thaliana*, *Oryza sativa*, *Triticum aestivum*; ^4^ characterized from *Arabidopsis thaliana*, *Physcomitrella patens*.
